# PPAR Ligands Function as Suppressors That Target Biological Actions of HMGB1

**DOI:** 10.1155/2016/2612743

**Published:** 2016-08-02

**Authors:** Shibo Ying, Xiang Xiao, Tianhui Chen, Jianlin Lou

**Affiliations:** ^1^Institute of Occupational Diseases, Zhejiang Academy of Medical Sciences, Hangzhou 310013, China; ^2^Department of Reproductive Physiology, Zhejiang Academy of Medical Sciences, Hangzhou 310013, China; ^3^Harvard T.H. Chan School of Public Health, Harvard University, Boston, MA 02115, USA

## Abstract

High mobility group box 1 (HMGB1), which has become one of the most intriguing molecules in inflammatory disorders and cancers and with which ligand-activated peroxisome proliferator-activated receptors (PPARs) are highly associated, is considered as a therapeutic target. Of particular interest is the fact that certain PPAR ligands have demonstrated their potent anti-inflammatory activities and potential anticancer effects. In this review article we summarize recent experimental evidence that PPAR ligands function as suppressors that target biological actions of HMGB1, including intracellular expression, receptor signaling cascades, and extracellular secretion of HMGB1 in cell lines and/or animal models. We also propose the possible mechanisms underlying PPAR involvement in inflammatory disorders and discuss the future therapeutic value of PPAR ligands targeting HMGB1 molecule for cancer prevention and treatment.

## 1. Introduction

### 1.1. PPARs and Their Ligand-Induced Activation

Peroxisome proliferator-activated receptors (PPARs) are members of the nuclear receptor family, with three isotypes PPAR-*α*, PPAR-*β*/*δ*, and PPAR-*γ*, which can be activated by specific PPAR ligands in cytoplasm. Ligand-activated PPARs transfer to nuclei and bind to the regulatory DNA elements located on the promoters of PPAR target genes, the peroxisome proliferator response elements (PPREs), thereby stimulating their transcription [1]. This mechanism is termed* transactivation* [2], and many PPAR target genes are found to be involved in metabolism and cell homeostasis [3, 4]. Furthermore, ligand-induced activation of PPARs also represses the expression of inflammatory response genes via a mechanism termed* ligand-dependent transrepression* [5]. In contrast to transactivation, transrepression does not involve binding of the nuclear receptor to its cognate DNA element but operates by antagonizing signal-dependent activation of PPAR target genes by other classes of transcription factors (TFs), including nuclear factor kappa B (NF-*κ*B) and activator protein-1 (AP-1), thereby reducing inflammatory reactions [1, 6].

In recent years, a number of natural or synthetic PPAR ligands, named agonists, have been identified in PPAR activation [2]. PPAR ligands contain a number of classes, such as eicosanoids, thiazolidinediones (TZDs), and fibrates [7]. A number of PPAR ligands are believed to have some pharmacological effects. For example, eicosapentaenoic acid (EPA), belonging to the eicosanoid class, is well known as a potent antioxidant with anti-inflammatory effects [8]. Several PPAR ligands from the TZD class are/were used as antidiabetic drugs, like troglitazone, rosiglitazone, and pioglitazone [1]. Fenofibrate is a drug of the fibrate class and mainly used to reduce cholesterol levels in patients at risk for cardiovascular disease [9]. In addition, telmisartan, a structurally unique angiotensin II receptor antagonist used for treatment of hypertension, is capable of functioning as an activator of PPAR-*γ* and PPAR-*β*/*δ* [10–12].

Although early studies focused on PPAR ligands in regulating cellular metabolism, there has been an increasing appreciation for PPARs' role in regulating a wide variety of biological processes, particularly inflammation and cancer [2]. PPARs function as cellular sensors, TFs, and inflammatory modulators. These findings have led to numerous applications, where PPARs' ligands serve as coadjuvants in protecting healthy tissue, anti-inflammatory treatment, and enhancing of anticancer therapies [13].

### 1.2. HMGB1 and Its Signaling Pathways

*Nuclear HMGB1*. High mobility group box 1 (HMGB1) is a member of the high mobility group superfamily, which exists ubiquitously in the nucleus and cytosol of mammalian cells. In normal state, HMGB1 is found as a DNA chaperone primarily in the nucleus, where it stabilizes nucleosomes and contributes to DNA transcription, replication, and recombination [14–16]. HMGB1 contains two DNA-binding HMG-box domains, which enable HMGB1 to bind different DNA structures without sequence-specificity [14]. Moreover, HMGB1 has been found to increase the binding affinity of many cancer-related TFs to their DNA-binding sites, such as NF-*κ*B, p53, p73, the retinoblastoma protein, and the estrogen receptor (ER) [14, 17], so that HMGB1 might modulate gene transcription driven by these TFs. However, whether HMGB1 plays a similar complex role in modulating gene expression by these TFs remains to be further explored. Furthermore, HMGB1 also has the ability to bend DNA and bind to nucleosomes at the dyad axis, which promotes nucleosome sliding and chromosomal stability [18].

*HMGB1 Secretion*. Interestingly, under certain physiological or pathological conditions, HMGB1 could translocate from the nucleus to the cytosol and then be released into the extracellular environment. Regarding its secretion, HMGB1 localization is dependent upon the acetylation of the lysine residues in its two nuclear localization signals (NLS1/2). Most nonacetylated HMGB1 is stored in the nucleus. Nevertheless, when nuclear HMGB1 is hyperacetylated, it mobilizes into the cytosol followed by active secretion into the extracellular space [19, 20]. In addition to this acetylation, phosphorylation [21–23] and methylation [24] of HMGB1 also contribute to the process of its secretion. HMGBl is currently believed to be secreted actively by inflammatory cells such as leukocytes and several tumor cells but also released passively during cell injury and death. 

*HMGB1-Mediated Signaling.* After secretion, the role of HMGB1 is dramatically changed. As a cytokine, extracellular HMGB1 stimulates several cell surface receptors, involving primarily the receptor for advanced glycation end-products (RAGE), the Toll-like family of receptors (TLRs), and chemokine receptor-4 (CXCR4) expressed on neighboring cells, and induces corresponding signal transduction pathways, resulting in activation of NF-*κ*B and mitogen-activated protein kinase (MAPK) pathways [14, 25, 26]. Moreover, the activation of the downstream MAPK pathways leads to the induction of AP-1, which also has a role in the expression of proinflammatory cytokines [26]. It is well known that both NF-*κ*B and AP-1 are inducible TFs that play a key role in the expression of a variety of genes involved in inflammation, cell survival, apoptosis, cell differentiation, and tumor genesis and progression [27–29]. Thus, the immune and oncogenic activities of HMGB1 have been assigned to its receptor signaling pathways in inflammation and carcinogenesis.

To the current knowledge, HMGB1's biological action is that it modulates immune and inflammatory responses and promotes cell proliferation, angiogenesis, and cell adhesion/migration [14]. As increasing evidence indicates, HMGB1 contributes to inflammation disorders and cancer development, and it is important that HMGB1 is considered as a novel therapeutic target [14, 16, 30].

## 2. HMGB1 Inhibition by PPAR Ligands in Inflammation

Recently, the role of PPARs and their ligands in mediating cellular responses to inflammation and cancers has been of particular interest. A number of studies provided new insights into the pleiotropic roles of PPAR ligands in suppressing the biological actions of HMGB1, including gene expression, signaling pathways, and extracellular release. Representative PPAR ligands and their effects on HMGB1 are outlined in Table 1. We described in this section the main findings from such studies regarding PPAR ligands, which may give a better understanding of the therapeutic value of PPAR ligands in the treatment of inflammation-related disorders.

### 2.1. Eicosapentaenoic Acid

Eicosapentaenoic acid (EPA) is an omega-3 fatty acid, which acts as a PPAR-*γ* agonist and prevents secondary rather than primary stroke [31, 32]. It is known that PPAR-*γ* is highly expressed in macrophages, endothelial cells, dendritic cells, T cells, and B cells [33–35]. Certain PPAR-*γ* agonists were also shown to modulate the central nervous and immune system by inhibiting the activation of microglia and astrocytes [36] and by modulating a proinflammatory mediator released from glial cells [37, 38]. One recent study from Japan found that EPA attenuated postischemic inflammation and brain damage in ovariectomized rats. Moreover, the increase of HMGB1 expression after cerebral ischemia activated inflammatory pathways via RAGE and TLRs, leading to brain damage [39]. The authors reported that EPA may exert both PPAR-*γ*-dependent and PPAR-*γ*-independent effects on the postischemic HMGB1/TLR9 pathway; that is, EPA regulated the HMGB1/TLR9 pathway bidirectionally in the postischemic cerebral cortex, and the downregulation of HMGB1 was PPAR-*γ*-independent, whereas the downregulation of RAGE and TLR9 was PPAR-*γ*-dependent. Consequently, upon the suppression of HMGB1 signaling, EPA may help to limit ischemic brain damage in postmenopausal women, in that the cortical infarct volume exacerbated by ovariectomization is associated with the upregulation of HMGB1/TLR9 pathway [39].

### 2.2. Telmisartan

Telmisartan is well known as angiotensin II blocker, used in the management of hypertension [40]. Moreover, telmisartan contains important structural components for PPAR-*γ* and exerts pleiotropic effects as a partial agonist [10]. A research group from Japan investigated the anti-inflammatory effects of telmisartan on middle cerebral artery occlusion in mice. According to their findings, telmisartan significantly decreases the number of macrophages/microglia cells expressing HMGB1 and downregulated plasma HMGB1 levels. Interestingly, the downregulation of plasma HMGB1 and the cerebroprotective effect induced by telmisartan were partially inhibited by PPAR-*γ* antagonist GW9662. As a result, telmisartan shows its cerebroprotective effect, which inhibits the inflammatory reactions after cerebral ischemia, in a PPAR-*γ*-dependent manner, targeting HMGB1 expression and secretion. Those findings suggest that telmisartan may be a potential treatment for postischemic injury [38].

### 2.3. Troglitazone

Troglitazone belongs to the thiazolidinedione class of drugs, which also includes rosiglitazone and pioglitazone described in the following sections. A group from China has reported that the activation of PPAR-*γ* by troglitazone inhibits HMGB1 expression at the transcription level in vascular endothelial cells, in which troglitazone interferes with NF-*κ*B or AP-1 signaling [41]. More specifically, troglitazone could inhibit not only the transcriptional activation of HMGB1 promoter, but also the activation of promoters driven by NF-*κ*B or AP-1 response elements. Furthermore, troglitazone modulated HMGB1 localization in the nucleus and cytoplasm. In activated immune cells, nuclear HMGB1 is modified by hyperacetylation or phosphorylation in response to certain stimuli as previously described in Section 1.2 [19, 21], which is the first critical step of the HMGB1 mobilization from the nucleus to the cytoplasm. The cytoplasmic HMGB1 is then concentrated into secretory lysosomes and subsequently secreted in response to inflammation.

Alternatively, another group in China recently reported that the activation of PPAR-*γ* by troglitazone inhibited HMGB1 protein expression through upregulation of microRNA- (miRNA-) 142-3p in THP-1 cells, a human monocytic cell line derived from an acute monocytic leukemia patient, and in murine peripheral blood mononuclear cells isolated from model mice blood [42]. This study indicated that ligand-activated PPAR-*γ* directly bound to the PPRE in the miRNA-142-3p promoter region, while PPAR-*γ*-induced miRNA-142-3p also could target the 3′-UTR of HMGB1 and thus suppress its expression* in vivo* [42].

These results suggest that troglitazone may regulate HMGB1 expression at both transcriptional and posttranscriptional level in PPAR-*γ*-mediated manner. Accordingly, these findings support the idea that troglitazone may be a therapeutic agent for suppressing excessive HMGB1 in inflammatory diseases.

### 2.4. Rosiglitazone

Rosiglitazone is another member of the thiazolidinedione class. Both rosiglitazone and pioglitazone were approved by the U.S. Food and Drug Administration in 1999. Despite their potent antidiabetic activity, their clinical use is associated with considerable adverse effects [49]. One report from Korea demonstrated that PPAR-*γ* activated by rosiglitazone was involved in the inhibition of lipopolysaccharide- (LPS-) induced HMGB1 release in RAW264.7 cells (macrophages from Abelson murine leukemia virus-induced tumor). The effect of rosiglitazone on HMGB1 secretion was abolished by the treatment with siRNA-PPAR-*γ* or PPAR-*γ* antagonist GW9662. In addition, rosiglitazone also inhibited LPS-induced expression of TRL-4 signal molecules, suggesting that rosiglitazone modulates HMGB1 release via a PPAR-*γ*-dependent mechanism on the HMGB1/TRL4 pathway [43]. Furthermore, they demonstrated that the inhibition of HMGB1 release by PPAR-*β*/*δ* and PPAR-*γ* was associated with a deacetylase enzyme, silent information regulator 1 (SIRT1). PPAR-mediated upregulation of SIRT1 modulates the status of HMGB1 acetylation, which is responsible for blockade of HMGB1 release in macrophages [50].

The inhibitory effect of rosiglitazone on HMGB1 was also described in a report from China [44]. In the pulmonary system, pretreatment of mice with rosiglitazone significantly suppressed LPS-induced acute lung injury (ALI) and reversed the elevated expression of HMGB1 and RAGE. The interaction of HMGB1 with RAGE activated the NF-*κ*B and MAPK pathways, resulting in upregulation of HMGB1, RAGE, and other proinflammatory mediators, thus promoting the development of ALI or acute respiratory distress syndrome (ARDS) [44, 45]. This study suggests that activation of PPAR-*γ* inhibits the development of LPS-induced inflammatory models by negative modulation of HMGB1 expression and its release.

### 2.5. Pioglitazone

Pioglitazone, one of the PPAR-*γ* agonists, is also from the thiazolidinedione class. One recent study found that pioglitazone inhibited the advanced glycation end-products- (AGEs-) induced elevation of HMGB1 protein expression in osteoarthritis (OA) chondrocytes [46]. AGEs may induce chondrocyte damage, which is important in the development of cartilage destruction and damage in age-related OA. In addition, it was reported that HMGB1 was involved in the pathogenesis of cartilage destruction in OA. Of note, cytoplasmic HMGB1-positive chondrocytes significantly increased in the deep layers of higher-grade cartilage [51]. On the other hand, previous studies indicated that PPAR-*γ* played a vital role in the development and progression of OA, and decreased expression of PPAR-*γ* in OA cartilage might lead to the increase of inflammatory response [52–54]. Interestingly, they found that AGEs induced inflammatory responses and downregulation of PPAR-*γ* expression via TLR-4 and RAGE in human OA chondrocytes [46]. As described in Section 1.2, both TLRs and RAGE are HMGB1 receptors on the cell surface. Hence, these results suggest that PPAR-*γ* agonist pioglitazone may inhibit both cytoplasmic HMGB1 and its cellular signaling. Accordingly, HMGB1 could be a potential target for pharmacologic intervention in the treatment of OA.

Similarly, another recent study indicated that pioglitazone might inhibit growth and invasion of human hepatocellular carcinoma (HCC) via blockade of the RAGE signaling [47]. Pioglitazone may also downregulate HMGB1 expression and inhibits HMGB1/RAGE pathway in SMMC-7721 and HepG2 cells [47]. RAGE is one of the main downstream receptors for HMGB1. In* in vitro* and* in vivo* HCC models, HMGB1 could induce cell proliferation, differentiation, cell death, angiogenesis, metastasis, and inflammation [55]. These findings gave a reasonable explanation for the antitumor activity of pioglitazone, at least in part. In addition, this study provides a novel insight into the therapeutic strategy targeting HMGB1 by PPAR ligands for human cancer, more than only for inflammatory disorders.

### 2.6. Fenofibrate

Fenofibrate, a PPAR-*α* agonist, has shown both protective effects against cardiac hypertrophy and inhibitory effects against inflammation [56]. In addition, more and more evidence has indicated that HMGB1 plays a crucial role in cardiovascular disease [57, 58]. Moreover, the decrease of nuclear HMGB1 was reported to be associated with human heart failure, and preserved amounts of nuclear HMGB1 could prevent cardiac hypertrophy [59]. One study from China provided experimental evidence that fenofibrate modulated basal and LPS-stimulated HMGB1 expression and localization in addition to its secretion in cardiomyocytes. Furthermore, fenofibrate protected the storage of the nuclear HMGB1 protein in the heart of mice and suppressed the development of cardiac hypertrophy induced by thoracic transverse aortic constriction [48]. These findings suggest that fenofibrate has inhibitory effects on HMGB1 expression and secretion in cardiomyocytes, which may restrain the development of cardiac hypertrophy.

## 3. Mechanisms of PPAR Ligands Action Targeting HMGB1

Although the above content in Section 2 demonstrated the inhibitory effects of several PPAR ligands on biological actions of HMGB1, it requires a comprehensive conclusion of the underlying molecular mechanisms. To the current knowledge, PPAR usually exists as a heterodimer complexed with retinoid X receptor (RXR); the complex is typically bound to corepressors. After ligand stimulation, the corepressor molecules are displaced, and PPAR, ligand, RXR, and coactivators form an active complex and transfer into the nucleus [60, 61]. In the nucleus, ligand-activated PPAR acts as a regulator at transcription level, in a termed PPAR-dependent manner. On the other hand, in a PPAR-independent manner, PPAR ligands work beyond that of PPAR pathways, mostly in the cytoplasm or extracellular environment.

**Figure 1:**
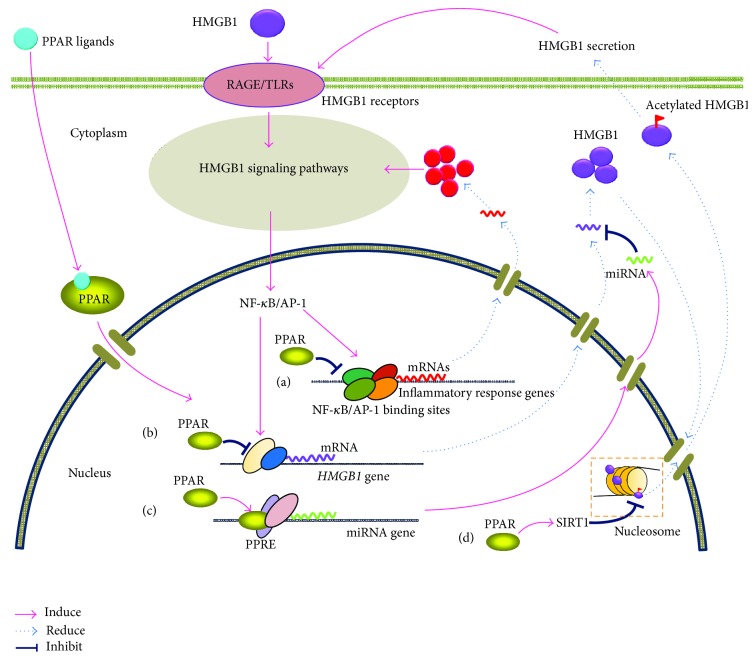
Schematic representation of PPAR ligands targeting HMGB1 via PPARs-mediated mechanisms. See details in text. PPAR: peroxisome proliferator-activated receptor; HMGB1: high mobility group box 1; TLR: Toll-like receptor; RAGE: receptor for advanced glycation end-products; PPRE: peroxisome proliferator response element; AP-1: activator protein-1; NF-*κ*B: nuclear factor-*κ*B; mRNA: messenger RNA; miRNA: microRNA; SIRT1: silent information regulator 1.

Indeed, eukaryotic gene expression, as well as protein function, is regulated at multiple levels, including epigenetic, transcriptional, posttranscriptional, translational, and posttranslational. Here, we outline the mechanisms of PPAR ligands' targeting of HMGB1 expression and biological actions at three levels. Despite one report suggesting that the pathway for the downregulation of HMGB1 by EPA is partly PPAR-*γ*-independent [39], most evidence supports the view that PPAR ligands function on HMGB1, the actions of which are mainly PPAR-dependent. To sum up, there are four possible routes as shown in Figure 1.

### 3.1. Transcriptional Level

*(a) Inhibition on HMGB1 Signaling Pathways*. PPAR ligands are capable of modulating HMGB1 biological actions at the transcriptional level in a PPAR-dependent manner. As reported, PPARs inhibit the activation of certain inflammatory response genes by binding TFs [5]; that is, they indirectly interfere with other TF pathways to inhibit transcription or* transrepression* [5]. Particularly, upon ligand stimulation, both PPAR-*α* and PPAR-*γ* can bind NF-*κ*B and AP-1 to repress NF-*κ*B and/or AP-1 target genes [62]. Cells expressing PPAR-*α* and PPAR-*γ* regulate inflammatory response genes by inhibiting NF-*κ*B and AP-1 pathways [3, 63], which are involved in HMGB1 signaling pathways. This mechanism decreases the levels of related molecules participating in an HMGB1-induced inflammatory response. Since downregulation of NF-*κ*B and AP-1 for PPARs has been found in different cell types including monocytes/macrophages, T lymphocytes, endothelial cells, smooth muscle cells, and keratinocytes [3, 64], there might be a general mechanism for the inhibition of inflammatory cytokines in various types of cells.

Several PPAR-*γ* ligands, such as troglitazone [41], rosiglitazone [44, 45], pioglitazone [46], ciglitazone [65], and ibuprofen [66], are found to play important roles in inhibition of TF NF-*κ*B or AP-1 activity. Also, PPAR ligands downregulate inflammatory cytokines, such as tumor necrosis factor- (TNF-) *α* [67], IL-2 [68], interferon- (IFN-) *γ* [68], IL-1*β* [67], IL-6 [67], MCP-1 [43], and MIP-1*β* [43]. Interestingly, HMGB1 transcription is upregulated by cytokines IFN-*γ* and TNF-*α* in matured THP-1 macrophages or human peripheral blood monocytes [69]. Secreted HMGB1 acts as a late proinflammatory mediator, but its release occurs considerably later than the secretion of classical early proinflammatory mediators, for examples, TNF-*α* and IL-1 [70]. Through this mechanism, PPAR ligands probably block inflammatory signaling cascades, leading to anti-inflammatory effects. Combined with the instances described in Section 2, much evidence supports this route of PPAR ligands targeting HMGB1-receptor signalings via PPARs-mediated mechanisms. 

*(b) TF-Mediated Transrepression*. Alternatively, it is likely that HMGB1 promoter-interfered direct inhibition by TF-mediated transrepression is also one possible route. The HMGB1 gene contains six exons and is located on the human chromosome 13q12 [71]. The human HMGB1 gene transcription is driven by a super strong TATA-less promoter, which may be one of the highest expressing mammalian promoters and exhibits an 18-fold greater transcriptional activity than that of the SV40 promoter in breast cancer cells [72]. This finding suggests that human HMGB1 gene is capable of expressing at an intensely high level. The basal expression level of HMGB1 observed in most cells is probably a result of repression of this strong transcriptional activity by an identified silencer in the proximal promoter [72]. It was reported that PPAR-*γ* ligand troglitazone could inhibit both transcriptional activity of the HMGB1 promoter and that of the NF-*κ*B or AP-1 in endothelial cells [41]. Furthermore, the failure to identify PPAR binding sites in the HMGB1 promoter suggests that the inhibitory effect is exerted by antagonizing the transcriptional activities of NF-*κ*B and AP-1, rather than through direct promoter-binding-mediated regulation by PPAR itself [41, 73–75].

Obviously, the two routes, (a) and (b), are related with each other grouped in transcriptional level, where both NF-*κ*B and AP-1 probably govern HMGB1 in a crosstalk way.

### 3.2. Posttranscriptional Level

*(c) miRNA-Mediated Regulation*. One recent study of troglitazone provided an unexpected potential route for PPAR ligand suppression of HMGB1 expression at the posttranscriptional level. This study reported that ligand-activated PPAR-*γ* inhibited HMGB1 expression through upregulation of miR-142-3p and suppressed inflammatory response in human acute monocytic leukemia THP-1 cell line [42]. Interestingly, this PPAR-*γ*-dependent inhibition effect on HMGB1 expression is involved in the upregulation of miR-142-3p targeting of the 3′-UTR of HMGB1 transcripts, by means of PPAR-*γ* binding directly to the PPRE in the miR-142-3p promoter region. This is considered to be a novel anti-inflammatory mechanism for PPAR-*γ* targeting of HMGB1 actions. In addition, it was previously demonstrated that miR-142-3p functioned as a potential tumor suppressor directly targeting HMGB1 in non-small-cell lung carcinoma cells [76]. PPREs consist of DNA-specific sequences, which are located in the promoter regions of its target genes. As a transcriptional activator, PPARs could directly bind to PPREs, thereby modulating the target genes at the transcriptional level (transactivation) [3]. In transactivation, PPARs directly regulate the transcription of target genes involved in lipid and lipoprotein metabolism, glucose homeostasis, and cell differentiation [3, 4]. However, there is some evidence that PPREs are also present in the miRNA promoter region, so that ligand-activated PPARs may regulate targets at the posttranscriptional level. For example, miR-145 was also found as a direct PPAR-*γ* transcriptional target* in vivo*. Rosiglitazone treatment induced PPAR-*γ* recruitment to the PPRE site, upstream of the miR-145 transcription start site, in colon cancer cell lines [77], though no direct association between miR-145 and HMGB1 transcript was noted in scientific literature. Altogether, miRNA-mediated regulation might be a possible nonclassical mechanism of ligand-activated PPAR targeting of HMGB1 at the posttranscriptional level.

### 3.3. Posttranslational Level

*(d) Suppressing HMGB1 Secretion.* Ligand-activated PPARs may also be involved in suppressing HMGB1 secretion rather than targeting HMGB1 in the extracellular space. In the normal state, via NLS1 and NLS2, synthesized HMGB1 is translocated from the cytosol of cells into the nucleus to bind DNA. As a nuclear nonhistone protein, HMGB1 is loosely and transiently associated with nucleosomes, which consist of tightly bound chromatin and DNA [69]. Upon the immunologic challenge, macrophages are activated, resulting in the forced hyperacetylation of NLS sites within HMGB1 in resting macrophages. This causes its relocalization to the cytosol, followed by packaging HMGB1 into secretory lysosomes, and releases it into the extracellular milieu [19, 78]. Of note, the acetylation of HMGB1 occurs in the nucleus; and this acetylation prevents HMGB1 from interacting with the nuclear-importer protein complex, so reentry to the nucleus is blocked [70]. Besides this acetylation, phosphorylation [21–23] and methylation [24] of HMGB1 are also shown to be related to its translocation during the secretion process in inflammatory or cancer cells. Therefore, posttranslational modifications of HMGB1 appear to be a critical step for its secretion. Recent studies have indicated that PPAR-*β*/*δ* and PPAR-*γ* are capable of suppressing the HMGB1 acetylation and thereby reducing the secretion of HMGB1 [43, 50]. In addition, PPAR-*γ* ligand telmisartan and rosiglitazone may inhibit HMGB1 release from macrophage cells [38, 43]. Notably, it is reported that acetylated HMGB1 is an effective substrate for SIRT1, which is NAD^+^-dependent class III protein deacetylase. Mediated by this deacetylase enzyme, ligand-activated PPAR-*β*/*δ* and PPAR-*γ* are able to upregulate SIRT1 expression in macrophages, resulting in deacetylation of HMGB1 protein. Hence, PPAR ligands also might suppress HMGB1 secretion through modulating the posttranslational modification status of HMGB1 in a PPAR-dependent mechanism.

## 4. Future Perspective of PPAR Ligands and HMGB1 in Cancers

Of particular interest is the possibility that ligand activation of PPARs may have potential anticancer effects, since both HMGB1 and PPARs are related to tumorigenesis and progression of cancers. In clinic, recent advances have shown that PPARs are potentially anticancer agents [13]; for example, in breast cancer, PPAR-*γ* is found mainly in well-differentiated and ER-positive breast carcinomas, modulates estrogenic actions, and has an inverse association with tumor size in humans [79]. This is similar to some inflammatory disorders, where both mRNA and protein levels of PPAR-*γ* are shown to be inversely correlated with levels of HMGB1 in peripheral blood mononuclear cells of patients with sepsis [42].

Also, some PPAR ligands have demonstrated their potent antitumor activity via PPAR-dependent and/or PPAR-independent mechanisms [80]. Of note, recent studies have revealed that PPAR-*γ* agonists exhibit antitumor activity and can induce apoptotic cell death in various malignant cell lineages, including malignant pleural mesothelioma, thyroid cancer, liposarcoma, breast adenocarcinoma, prostate carcinoma, colorectal carcinoma, non-small-cell lung carcinoma, pancreatic carcinoma, bladder cancer, and gastric carcinoma [80–82].

After its discovery in 1973 [83], the multifaceted HMGB1 has recently been identified as an important mediator of cancers, which initiated a new field of translational medicine that targets HMGB1 [14]. HMGB1 is involved in tumor development, proliferation, invasion, and metastasis, and its high levels are associated with a poor clinical prognosis [84–87]. The involvement of HMGB1 in cancer is complicated, and nuclear/intracellular and extracellular forms of HMGB1 have been implicated in tumor formation, progression, and metastasis and in response to chemotherapeutics. Elevated expression of HMGB1 occurs in several solid tumors, including melanoma, colon cancer, prostate cancer, pancreatic cancer, breast cancer, and malignant mesothelioma [85–88].

The underlying mechanisms of HMGB1-receptor signaling involving PPAR ligands remain to be explored. A few pervious researches on some cell lines and/or in animal models have already revealed their close relationship (Table 1). These studies showed that PPAR ligands not only suppress HMGB1 signaling pathways, but also inhibit HMGB1 gene expression and secretion. Therefore, additional information about signal transduction pathways and potential diagnostic utility is warranted. In view of the PPAR ligands interaction with HMGB1, taking into account potential target for molecular therapy, it is crucial to investigate the anticancer mechanisms not yet identified in comprehensive pathways at transcriptional, posttranscriptional, translational, and posttranslational levels. This may pave the way for HMGB1 utilization as a therapeutic target for inflammatory disorders and related cancers in the future. Conversely, advancement of the understanding of the anticancer mechanism of PPARs will greatly improve utilization of PPAR ligands for treatment of cancers or cancer-related diseases.

## Figures and Tables

**Table 1:** PPAR ligands suppressing biological actions of HMGB1.

PPAR ligands	PPAR types	Possible involved HMGB1 signaling or expression	Effects on biological actions of HMGB1	Cell lines	Animal model	Potential applied diseases	References
EPA	PPAR-*γ*	HMGB1/TLR9 signaling	EPA inhibits HMGB1/TLR9 pathway and downregulates HMGB1 expression in brain cortex.	—	Ovariectomized rat model of cerebral ischemia	Ischemic brain damage and ischemic stroke	[39]

Telmisartan	PPAR-*γ*	—	Telmisartan decreases plasma HMGB1 levels and suppresses the expression of HMGB1 in macrophages or microglial cells.	—	Mice model of focal cerebral ischemia	Postischemic injury	[38]

Troglitazone	PPAR-*γ*	Transcriptional activity of HMGB1 promoter and NF-*κ*B or AP-1 signaling	Troglitazone inhibits HMGB1 expression in endothelial cells.	Vascular endothelial cells	—	Sepsis, arthritis, and atherosclerosis	[41]
		miRNA-based regulation	Troglitazone inhibits HMGB1 expression through upregulation of miR-142-3p.	THP-1 cells	Mice model of endotoxemia	Sepsis	[42]

Rosiglitazone	PPAR-*γ*	HMGB1/TLR4 signaling	Rosiglitazone decreases LPS-induced plasma HMGB1 levels and inhibits HMGB1 release of RAW264.7 cells.	RAW264.7 cells	Mice model of endotoxemia	Sepsis	[43]
		HMGB1/RAGE signaling	Rosiglitazone reverses LPS-induced elevation of HMGB1 in bronchoalveolar lavage fluid.	—	Mice model of ALI	ALI and ARDS	[44, 45]

Pioglitazone	PPAR-*γ*	HMGB1/TLR4 and HMGB1/RAGE signaling	Pioglitazone reverses AGEs-induced elevation of HMGB1 expression in OA chondrocytes.	Chondrocytes	—	OA	[46]
		HMGB1/RAGE signaling	Pioglitazone downregulates HMGB1 expression and inhibits HMGB1/RAGE signaling in HCC cells.	SMMC-7721 and HepG2 cells	—	Hepatocellular carcinoma	[47]

Fenofibrate	PPAR-*α*	—	Fenofibrate reverses basal and LPS-induced HMGB1 expression, as well as modulating its cellular localization.	Cardiomyocytes	Mice model of hypertrophic myocardium	Cardiac hypertrophy	[48]

EPA: eicosapentaenoic acid; LPS: lipopolysaccharide; TLR: Toll-like receptor; RAGE: receptor for advanced glycation end-products; ALI: acute lung injury; ARDS: acute respiratory distress syndrome; AGEs: advanced glycation end-products; OA: osteoarthritis; HCC: hepatocellular carcinoma.

## References

[1] Cariou B., Charbonnel B., Staels B. (2012). Thiazolidinediones and PPAR*γ* agonists: time for a reassessment. *Trends in Endocrinology and Metabolism*.

[2] Croasdell A., Duffney P. F., Kim N., Lacy S. H., Sime P. J., Phipps R. P. (2015). PPAR*γ* and the innate immune system mediate the resolution of inflammation. *PPAR Research*.

[3] Blanquart C., Barbier O., Fruchart J. C., Staels B., Glineur C. (2003). Peroxisome proliferator-activated receptors: regulation of transcriptional activities and roles in inflammation. *Journal of Steroid Biochemistry and Molecular Biology*.

[4] Duez H., Fruchart J.-C., Staels B. (2001). PPARs in inflammation, atherosclerosis and thrombosis. *Journal of Cardiovascular Risk*.

[5] Chinetti G., Fruchart J. C., Staels B. (2003). Peroxisome proliferator-activated receptors and inflammation: from basic science to clinical applications. *International Journal of Obesity*.

[6] Straus D. S., Glass C. K. (2007). Anti-inflammatory actions of PPAR ligands: new insights on cellular and molecular mechanisms. *Trends in Immunology*.

[7] Kroker A. J., Bruning J. B. (2015). Review of the structural and dynamic mechanisms of PPAR*γ* partial agonism. *PPAR Research*.

[8] Kohashi K., Nakagomi A., Saiki Y. (2014). Effects of eicosapentaenoic acid on the levels of inflammatory markers, cardiac function and long-term prognosis in chronic heart failure patients with dyslipidemia. *Journal of Atherosclerosis and Thrombosis*.

[9] Wong T. Y., Simó R., Mitchell P. (2012). Fenofibrate—a potential systemic treatment for diabetic retinopathy?. *American Journal of Ophthalmology*.

[10] Goebel M., Clemenz M., Staels B., Unger T., Kintscher U., Gust R. (2009). Characterization of new PPAR*γ* agonists: analysis of telmisartan's structural components. *ChemMedChem*.

[11] Li L., Luo Z., Yu H. (2013). Telmisartan improves insulin resistance of skeletal muscle through peroxisome proliferator-activated receptor-*δ* activation. *Diabetes*.

[12] Funao K., Matsuyama M., Kawahito Y. (2009). Telmisartan as a peroxisome proliferator-activated receptor-*γ* ligand is a new target in the treatment of human renal cell carcinoma. *Molecular Medicine Reports*.

[13] Robbins M. E. C., Linard C., Panigrahy D. (2010). PPARs and anticancer therapies. *PPAR Research*.

[14] Kang R., Zhang Q., Zeh H. J., Lotze M. T., Tang D. (2013). HMGB1 in cancer: good, bad, or both?. *Clinical Cancer Research*.

[15] Naglova H., Bucova M. (2012). HMGB1 and its physiological and pathological roles. *Bratislavské Lekárske Listy*.

[16] Sims G. P., Rowe D. C., Rietdijk S. T., Herbst R., Coyle A. J. (2010). HMGB1 and RAGE in inflammation and cancer. *Annual Review of Immunology*.

[17] Agresti A., Lupo R., Bianchi M. E., Müller S. (2003). HMGB1 interacts differentially with members of the Rel family of transcription factors. *Biochemical and Biophysical Research Communications*.

[18] Travers A. A. (2003). Priming the nucleosome: a role for HMGB proteins?. *EMBO Reports*.

[19] Bonaldi T., Talamo F., Scaffidi P. (2003). Monocytic cells hyperacetylate chromatin protein HMGB1 to redirect it towards secretion. *The EMBO Journal*.

[20] Lu B., Antoine D. J., Kwan K. (2014). JAK/STAT1 signaling promotes HMGB1 hyperacetylation and nuclear translocation. *Proceedings of the National Academy of Sciences of the United States of America*.

[21] Youn J. H., Shin J.-S. (2006). Nucleocytoplasmic shuttling of HMGB1 is regulated by phosphorylation that redirects it toward secretion. *The Journal of Immunology*.

[22] Oh Y. J., Youn J. H., Ji Y. (2009). HMGB1 is phosphorylated by classical protein kinase C and is secreted by a calcium-dependent mechanism. *The Journal of Immunology*.

[23] Kang H. J., Lee H., Choi H.-J. (2009). Non-histone nuclear factor HMGB1 is phosphorylated and secreted in colon cancers. *Laboratory Investigation*.

[24] Ito I., Fukazawa J., Yoshida M. (2007). Post-translational methylation of high mobility group box 1 (HMGB1) causes its cytoplasmic localization in neutrophils. *The Journal of Biological Chemistry*.

[25] Park J. S., Svetkauskaite D., He Q. (2004). Involvement of toll-like receptors 2 and 4 in cellular activation by high mobility group box 1 protein. *The Journal of Biological Chemistry*.

[26] Nogueira-Machado J. A., Volpe C. M., Veloso C. A., Chaves M. M. (2011). HMGB1, TLR and RAGE: a functional tripod that leads to diabetic inflammation. *Expert Opinion on Therapeutic Targets*.

[27] Naugler W. E., Karin M. (2008). NF-*κ*B and cancer—identifying targets and mechanisms. *Current Opinion in Genetics & Development*.

[28] Hess J., Angel P., Schorpp-Kistner M. (2004). AP-1 subunits: quarrel and harmony among siblings. *Journal of Cell Science*.

[29] Ameyar M., Wisniewska M., Weitzman J. B. (2003). A role for AP-1 in apoptosis: the case for and against. *Biochimie*.

[30] Tang D., Kang R., Zeh H. J., Lotze M. T. (2010). High-mobility group box 1 and cancer. *Biochimica et Biophysica Acta-Gene Regulatory Mechanisms*.

[31] Tanaka K., Ishikawa Y., Yokoyama M. (2008). Reduction in the recurrence of stroke by eicosapentaenoic acid for hypercholesterolemic patients: subanalysis of the JELIS trial. *Stroke*.

[32] Ulu A., Harris T. R., Morisseau C. (2013). Anti-inflammatory effects of *ω*-3 polyunsaturated fatty acids and soluble epoxide hydrolase inhibitors in angiotensin-II-dependent hypertension. *Journal of Cardiovascular Pharmacology*.

[33] Clark R. B., Bishop-Bailey D., Estrada-Hernandez T., Hla T., Puddington L., Padula S. J. (2000). The nuclear receptor PPAR*γ* and immunoregulation: PPAR*γ* mediates inhibition of helper T cell responses. *Journal of Immunology*.

[34] Greene M. E., Blumberg B., McBride O. W. (1995). Isolation of the human peroxisome proliferator activated receptor gamma cDNA: expression in hematopoietic cells and chromosomal mapping. *Gene Expression*.

[35] Marx N., Sukhova G., Murphy C., Libby P., Plutzky J. (1998). Macrophages in human atheroma contain PPAR*γ*: differentiation-dependent peroxisomal proliferator-activated receptor *γ* (PPAR*γ*) expression and reduction of MMP-9 activity through PPAR*γ* activation in mononuclear phagocytes *in vitro*. *The American Journal of Pathology*.

[36] Storer P. D., Xu J., Chavis J., Drew P. D. (2005). Peroxisome proliferator-activated receptor-gamma agonists inhibit the activation of microglia and astrocytes: implications for multiple sclerosis. *Journal of Neuroimmunology*.

[37] Gurley C., Nichols J., Liu S., Phulwani N. K., Esen N., Kielian T. (2008). Microglia and astrocyte activation by toll-like receptor ligands: modulation by PPAR-*γ* Agonists. *PPAR Research*.

[38] Haraguchi T., Takasaki K., Naito T. (2009). Cerebroprotective action of telmisartan by inhibition of macrophages/microglia expressing HMGB1 via a peroxisome proliferator-activated receptor *γ*-dependent mechanism. *Neuroscience Letters*.

[39] Sumiyoshi M., Satomi J., Kitazato K. T. (2015). PPAR*γ*-dependent and -independent inhibition of the HMGB1/TLR9 pathway by eicosapentaenoic acid attenuates ischemic brain damage in ovariectomized rats. *Journal of Stroke and Cerebrovascular Diseases*.

[40] Sharpe M., Jarvis B., Goa K. L. (2001). Telmisartan: a review of its use in hypertension. *Drugs*.

[41] Gao M., Hu Z., Zheng Y. (2011). Peroxisome proliferator-activated receptor *γ* agonist troglitazone inhibits high mobility group box 1 expression in endothelial cells via suppressing transcriptional activity of nuclear factor *κ*b and activator protein 1. *Shock*.

[42] Yuan Z., Luo G., Li X., Chen J., Wu J., Peng Y. (2016). PPAR*γ* inhibits HMGB1 expression through upregulation of miR-142-3p in vitro and in vivo. *Cellular Signalling*.

[43] Hwang J. S., Kang E. S., Ham S. A. (2012). Activation of peroxisome proliferator-activated receptor *γ* by rosiglitazone inhibits lipopolysaccharide-induced release of high mobility group box 1. *Mediators of Inflammation*.

[44] Wang G., Liu L., Zhang Y. (2014). Activation of PPAR*γ* attenuates LPS-induced acute lung injury by inhibition of HMGB1-RAGE levels. *European Journal of Pharmacology*.

[45] Wang G., Han D., Zhang Y. (2013). A novel hypothesis: up-regulation of HO-1 by activation of PPAR*γ* inhibits HMGB1-RAGE signaling pathway and ameliorates the development of ALI/ARDS. *Journal of Thoracic Disease*.

[46] Chen Y. J., Sheu M. L., Tsai K. S., Yang R. S., Liu S. H. (2013). Advanced glycation end products induce peroxisome proliferator-activated receptor *γ* down-regulation-related inflammatory signals in human chondrocytes via Toll-like receptor-4 and receptor for advanced glycation end products. *PLoS ONE*.

[47] Yang Y., Zhao L.-H., Huang B. (2015). Pioglitazone, a PPAR*γ* agonist, inhibits growth and invasion of human hepatocellular carcinoma via blockade of the rage signaling. *Molecular Carcinogenesis*.

[48] Jia Z., Xue R., Liu G. (2014). HMGB1 is involved in the protective effect of the PPAR*α* agonist fenofibrate against cardiac hypertrophy. *PPAR Research*.

[49] Chigurupati S., Dhanaraj S. A., Balakumar P. (2015). A step ahead of PPAR*γ* full agonists to PPAR*γ* partial agonists: therapeutic perspectives in the management of diabetic insulin resistance. *European Journal of Pharmacology*.

[50] Hwang J. S., Lee W. J., Kang E. S. (2014). Ligand-activated peroxisome proliferator-activated receptor-*δ* and -*γ* inhibit lipopolysaccharide-primed release of high mobility group box 1 through upregulation of SIRT1. *Cell Death and Disease*.

[51] Terada C., Yoshida A., Nasu Y. (2011). Gene expression and localization of high-mobility group box chromosomal protein-1 (HMGB-1) in human osteoarthritic cartilage. *Acta Medica Okayama*.

[52] Fahmi H., Martel-Pelletier J., Pelletier J.-P., Kapoor M. (2011). Peroxisome proliferator-activated receptor gamma in osteoarthritis. *Modern Rheumatology*.

[53] Ma C., Zhang Y., Li Y.-Q., Chen C., Cai W., Zeng Y.-L. (2015). The role of PPAR*γ* in advanced glycation end products-induced inflammatory response in human chondrocytes. *PLoS ONE*.

[54] Afif H., Benderdour M., Mfuna-Endam L. (2007). Peroxisome proliferator-activated receptor *γ*1 expression is diminished in human osteoarthritic cartilage and is downregulated by interleukin-1*β* in articular chondrocytes. *Arthritis Research and Therapy*.

[55] Wang X., Xiang L., Li H. (2015). The role of HMGB1 signaling pathway in the development and progression of hepatocellular carcinoma: a review. *International Journal of Molecular Sciences*.

[56] Ichihara S., Obata K., Yamada Y. (2006). Attenuation of cardiac dysfunction by a PPAR-*α* agonist is associated with down-regulation of redox-regulated transcription factors. *Journal of Molecular and Cellular Cardiology*.

[57] Li W., Sama A. E., Wang H. (2006). Role of HMGB1 in cardiovascular diseases. *Current Opinion in Pharmacology*.

[58] de Souza A. W. S., Westra J., Limburg P. C., Bijl M., Kallenberg C. G. M. (2012). HMGB1 in vascular diseases: its role in vascular inflammation and atherosclerosis. *Autoimmunity Reviews*.

[59] Funayama A., Shishido T., Netsu S. (2013). Cardiac nuclear high mobility group box 1 prevents the development of cardiac hypertrophy and heart failure. *Cardiovascular Research*.

[60] Berger J., Moller D. E. (2002). The mechanisms of action of PPARs. *Annual Review of Medicine*.

[61] Coll T., Rodríguez-Calvo R., Barroso E. (2009). Peroxisome proliferator-activated receptor (PPAR) *β*/*δ*: a new potential therapeutic target for the treatment of metabolic syndrome. *Current Molecular Pharmacology*.

[62] Harmon G. S., Lam M. T., Glass C. K. (2011). PPARs and lipid ligands in inflammation and metabolism. *Chemical Reviews*.

[63] Olefsky J. M., Glass C. K. (2009). Macrophages, inflammation, and insulin resistance. *Annual Review of Physiology*.

[64] Schmuth M., Moosbrugger-Martinz V., Blunder S., Dubrac S. (2014). Role of PPAR, LXR, and PXR in epidermal homeostasis and inflammation. *Biochimica et Biophysica Acta (BBA)—Molecular and Cell Biology of Lipids*.

[65] Wang P., Anderson P. O., Chen S., Paulsson K. M., Sjögren H., Li S. (2001). Inhibition of the transcription factors AP-1 and NF-*κ*B in CD4 T cells by peroxisome proliferator-activated receptor *γ* ligands. *International Immunopharmacology*.

[66] Tegeder I., Pfeilschifter J., Geisslinger G. (2001). Cyclooxygenase-independent actions of cyclooxygenase inhibitors. *The FASEB Journal*.

[67] Jiang C., Ting A. T., Seed B. (1998). PPAR-*γ* agonists inhibit production of monocyte inflammatory cytokines. *Nature*.

[68] van Neerven S., Kampmann E., Mey J. (2008). RAR/RXR and PPAR/RXR signaling in neurological and psychiatric diseases. *Progress in Neurobiology*.

[69] Ellerman J. E., Brown C. K., de Vera M. (2007). Masquerader: high mobility group box-1 and cancer. *Clinical Cancer Research*.

[70] Lotze M. T., Tracey K. J. (2005). High-mobility group box 1 protein (HMGB1): nuclear weapon in the immune arsenal. *Nature Reviews Immunology*.

[71] Ferrari S., Finelli P., Rocchi M., Bianchi M. E. (1996). The active gene that encodes human high mobility group 1 protein (HMG1) contains introns and maps to chromosome 13. *Genomics*.

[72] Lum H. K., Lee K.-L. (2001). The human HMGB1 promoter is modulated by a silencer and an enhancer-containing intron. *Biochimica et Biophysica Acta—Gene Structure and Expression*.

[73] Szanto A., Nagy L. (2008). The many faces of PPAR*γ*: anti-inflammatory by any means?. *Immunobiology*.

[74] Duan S. Z., Usher M. G., Mortensen R. M. (2009). PPARs: the vasculature, inflammation and hypertension. *Current Opinion in Nephrology and Hypertension*.

[75] Duan S. Z., Usher M. G., Mortensen R. M. (2008). Peroxisome proliferator-activated receptor-*γ*-mediated effects in the vasculature. *Circulation Research*.

[76] Xiao P., Liu W. L. (2015). MiR-142-3p functions as a potential tumor suppressor directly targeting HMGB1 in non-small-cell lung carcinoma. *International Journal of Clinical and Experimental Pathology*.

[77] Panza A., Votino C., Gentile A. (2014). Peroxisome proliferator-activated receptor *γ*-mediated induction of microRNA-145 opposes tumor phenotype in colorectal cancer. *Biochimica et Biophysica Acta (BBA)—Molecular Cell Research*.

[78] Gardella S., Andrei C., Ferrera D. (2002). The nuclear protein HMGB1 is secreted by monocytes via a non-classical, vesicle-mediated secretory pathway. *The EMBO Reports*.

[79] Suzuki T., Hayashi S., Miki Y. (2006). Peroxisome proliferator-activated receptor *γ* in human breast carcinoma: a modulator of estrogenic actions. *Endocrine-Related Cancer*.

[80] Benedetti E., Galzio R., D'Angelo B., Cer M. P., Cimini A. (2010). PPARs in human neuroepithelial tumors: PPAR ligands as anticancer therapies for the most common human neuroepithelial tumors. *PPAR Research*.

[81] Hamaguchi N., Hamada H., Miyoshi S. (2010). *In vitro* and *in vivo* therapeutic efficacy of the PPAR-*γ* agonist troglitazone in combination with cisplatin against malignant pleural mesothelioma cell growth. *Cancer Science*.

[82] Ferrari S. M., Materazzi G., Baldini E. (2016). Antineoplastic effects of PPARgamma agonists, with a special focus on thyroid cancer. *Current Medicinal Chemistry*.

[83] Goodwin G. H., Sanders C., Johns E. W. (1973). A new group of chromatin associated proteins with a high content of acidic and basic amino acids. *European Journal of Biochemistry*.

[84] Tabata C., Shibata E., Tabata R. (2013). Serum HMGB1 as a prognostic marker for malignant pleural mesothelioma. *BMC Cancer*.

[85] Paek J., Lee M., Nam E. J., Kim S. W., Kim Y. T. (2016). Clinical impact of high mobility group box 1 protein in epithelial ovarian cancer. *Archives of Gynecology and Obstetrics*.

[86] Sun S., Zhang W., Cui Z. (2015). High mobility group box-1 and its clinical value in breast cancer. *OncoTargets and Therapy*.

[87] Wang H., Li Z., Sun Y. (2015). Relationship between high-mobility group box 1 overexpression in ovarian cancer tissue and serum: a meta-analysis. *OncoTargets and Therapy*.

[88] Tabata C., Kanemura S., Tabata R. (2013). Serum HMGB1 as a diagnostic marker for malignant peritoneal mesothelioma. *Journal of Clinical Gastroenterology*.

